# *In Vitro* Screening for Acetylcholinesterase Inhibition and Antioxidant Activity of *Quercus suber* Cork and Corkback Extracts

**DOI:** 10.1155/2020/3825629

**Published:** 2020-07-19

**Authors:** Joana Ferreira, Sara Santos, Helena Pereira

**Affiliations:** Centro de Estudos Florestais, Instituto Superior de Agronomia, Universidade de Lisboa, Lisbon, Portugal

## Abstract

**Purpose:**

Acetylcholinesterase (AChE) inhibitors are used to treat Alzheimer's patients because they enhance cholinergic neurotransmission. It is urgent to find new and efficient inhibitors from natural sources, highly bioavailable with low or no toxicity. The plant kingdom is extremely rich in a variety of compounds that are potent AChE inhibitors: flavonoids and other phenolic compounds have been recognized as promising Alzheimer's treatment agents. In this study, *in vitro* acetylcholinesterase inhibition, antioxidant activities, and total flavonoid and phenolic contents of ethanol-water extracts from *Quercus suber* cork and corkback were evaluated.

**Methods:**

The acetylcholinesterase activity was determined by a colorimetric assay based on Ellman's methodology. The Folin–Ciocalteu colorimetric method was used for total phenolic content determination and the aluminium chloride method for the determination of total flavonoid content. Antioxidant activity assays were performed using the DPPH and FRAP assays.

**Results:**

The acetylcholinesterase inhibitory activity from *Q. suber* cork and corkback ethanol-water extracts was as follows: 62% inhibition with corkback extracts over 0.5 mg/mL and around 49% inhibition in cork extracts over 1.0 mg/mL extracts' concentration. Regarding the DPPH radical scavenging activity, the concentrations of cork and corkback ethanol-water extracts required for 50% DPPH inhibition (IC50) were 3.2 *μ*g/mL and 4.0–5.2 *μ*g/mL. Corkback extracts are less effective than Trolox standard (3.2 *μ*g/mL) but cork extracts showed the same free radical scavenging activity compared to Trolox. Cork and corkback extracts have antioxidant power of 750.9–775.4 mg TEAC/g extract and 1051.2–2052.4 mg TEAC/g extract, respectively, which are significantly higher than the ones obtained with Trolox: 19.6–21.0 mg TEAC/g extract (cork assays) and 57.4–66.3 mg TEAC/g extract (corkback assays). The amounts of total phenolic (TPC) and flavonoid (TFC) compounds were 8.7–32.3 mg GAE/g and 4.8–10.7 mg CE/g dry mass for cork and 5.4–5.7 mg GAE/g and 42.5 mg CE/g dry mass for corkback extracts, respectively, using catechin (CE) and GAE (gallic acid) as standards.

**Conclusion:**

These findings demonstrate the remarkable potential of these extracts as valuable source of antioxidants with interesting acetylcholinesterase inhibitory activity.

## 1. Introduction

Medicinal plants are used by a large share of the world population as a source of therapeutic agents for their primary health care [1–3]. Some secondary metabolites of plants are bioactive compounds used for the treatment of several diseases, e.g., to fight the damage caused by reactive oxygen species resulting in several human pathologies such as arthritis, cancer, inflammatory conditions, or heart disease [4, 5]. Sources of free radicals involved in this oxidative stress are chemical products, toxins, radiation, pollution, agriculture toxics, and preservatives used in foods [6].

Phenols and flavonoids may have the potential to function as antioxidants by scavenging free radicals via hydrogenation or complexation with oxidizing species due to their conjugated ring structures and hydroxyl groups [6]. In the recent decades, natural products and drugs derived from natural products have become a research focus to search for naturally occurring antioxidants to be used in food, cosmetics, pharmaceutics, and medicinal products [7].

Among several neurological disorders, Alzheimer's disease (AD) is a progressive neurodegenerative disorder and the most prevalent cause of dementia in elderly people (in 2019 ADI estimated that there are over 50 million people living with dementia globally) [8, 9]. Although not fully understood, the “cholinergic hypothesis” proposes that the neurodegenerative mechanism is a decline of the neurotransmitter acetylcholine in the brain. The drugs presently used to treat AD patients act as enhancers of the acetylcholine level in the brain, responsible for central cholinergic transmission [10]. After being delivered across neuronal synapses, acetylcholine is hydrolyzed to a choline and an acetyl group by the enzyme acetylcholinesterase [8]. Therefore, applying inhibitors of acetylcholinesterase, acetylcholine is not hydrolyzed, maintaining its activity as a neurotransmitter [11, 12]. However, some of the drugs approved for the treatment of AD symptoms show hepatotoxicity [12], higher risk of urinary incontinence [13], increased risk of bradycardia and other cardiovascular adverse effects [14], pulmonary disorders [15], and involuntary weight loss [16], and the search continues for new drugs that are more efficient in brain penetration, less harmful, and with high bioavailability [12].

The acetylcholinesterase enzyme (AChE) is an attractive target for the discovery of mechanism-based inhibitors because of its role in hydrolysis of the neurotransmitter acetylcholine. AChE inhibitors as rivastigmine [17], donepezil, or galantamine [18] are currently the most effective agents to treat cognitive symptoms of AD and have other possible therapeutic applications in the treatment of other neurodegenerative disorders. Recently, research on prevention and treatment of Alzheimer has focused on naturally occurring acetylcholinesterase (AChE) inhibitors from plants, namely, polyphenolic compounds such as flavonoids with inhibitory capacity similar to that of the currently prescribed AChE inhibitor drugs [17–22], of which the alkaloid galantamine is a perfect example [18]. This compound offers the advantage of being better tolerated and cheaper, since it is commonly found in foods, and its antioxidant activity and strong metal chelator capability may also contribute to the decrease of the oxidative stress that affects tissues associated with Alzheimer's disease [8].

This study considers cork, the outer bark from *Quercus suber,* a potential natural source of bioactive compounds. Cork has a high proportion of lipophilic and polar metabolites [23]. The detailed composition of cork lipophilic extracts has been investigated, showing that they are mainly composed by aliphatics, phenolics, and triterpenes [24]. The information available on the ethanol and water extracts is scarce, but they are rich in phenolic compounds and have relatively high antioxidant activity as radical scavengers [25]. The cork material has peculiar and unique properties such as high elasticity and low permeability that allows several applications, such as wine stoppers and thermal or acoustic insulators, thereby building up an economically relevant cork industry [26]. The residues produced by the cork-based industries may be an inexpensive source of substances with useful chemical characteristics and properties.

The aim of this study is to investigate the ethanol-water extracts of cork and of the corkback residues as prospective new sources of compounds with both antioxidant and acetylcholinesterase inhibitory activity that could potentially be applied in the treatment of neurodegenerative disorders such as AD.

## 2. Materials and Methods

### 2.1. Plant Material

Cork and corkback samples were obtained from *Quercus suber* collected in Herdade da Contenda (Moura, Portugal) and made available as planks by a cork processing unit from Amorins&Irmãos situated in Lordelo, Santa Maria da Feira, Portugal.

The cork and corkback samples were separated manually with a scalpel and then air-dried in controlled indoor conditions regarding humidity and temperature, in the absence of light. The corkback is the outer layer of the cork plank; it is the phloemic layer that remains to the outside when the underlying periderm is formed and the cork layer produced (Pereira [26]).

The samples were ground individually in a cutting mill (Retsch SM 2000) using an output sieve with 10 mm × 10 mm openings, followed by a second pass with a 2 mm × 2 mm output sieve, and then fractionated with a vibratory system (Retsch AS 200basic) with standard sieves with the following mesh sizes: 80 (0.180 mm), 60 (0.250 mm), 40 (0.425 mm), 20 (0.850 mm), and 15 (1 mm). After sieving, the 2.0–1.0 mm and 0.45–0.25 mm fractions were collected for chemical analysis.

Fractioning of cork and corkback samples was done in triplicate.

### 2.2. Chemicals

The following chemicals were supplied by Sigma-Aldrich (St. Louis, MO, USA): dichloromethane, ethanol, gallic acid (GA), Folin–Ciocalteu reagent, sodium carbonate (Na_2_CO_3_), catechin (CA), sodium hydroxide (NaOH), sodium nitrate (NaNO_2_), aluminium chloride (AlCl_3_), 2,2-diphenyl-1-picryhydrazyl (DPPH), 2,4,6-tripyridyl-s-triazine (TPTZ), sodium acetate (NaOCH_3_), FeCl_3_.6H_2_O, 6-hydroxy-2,5,7,8-tetramethylchroman-2-carboxylic acid (Trolox), acetylcholinesterase (AChE) from electric eel (type VI-S 349 U/mg solid, 411 mg/U protein), 5,5′-dithio-bis-[2-nitrobenzoic acid] (DTNB), and substrate acetylthiocholine iodide (AChI). For the preparation of buffer, dipotassium hydrogen phosphate (K_2_HPO_4_) and potassium hydroxide (KOH) both from Acros Organics (Pittsburgh, PA, USA) were used (extra pure analytical grade).

### 2.3. Preparation of Extract Solutions

**Figure 1:**
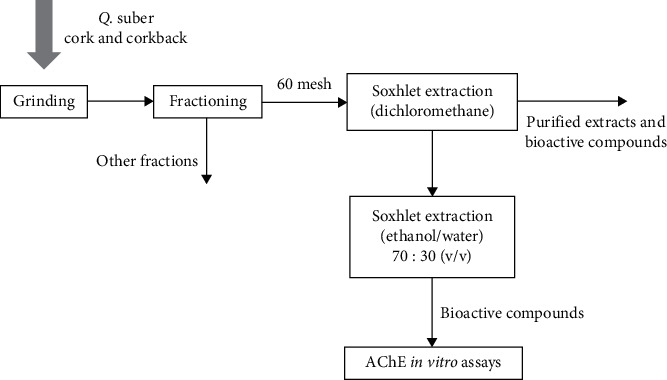
Scheme of the followed methodology towards the *in vitro* assays with AChE.

The fractionated cork and corkback samples were first submitted to a successive extraction in a Soxhlet apparatus, using dichloromethane for 6 h. Ethanol-water extracts were obtained using an ethanol-water solvent solution 70 : 30 (v : v) by the same methodology, during 48 h (Figure 1).

### 2.4. Total Phenolic and Total Flavonoid Contents

The total phenolic content (TPC) of the ethanol-water extracts of cork and corkback samples was determined using a modified Folin–Ciocalteu colorimetric method [27], in which a phosphowolframate-phosphomolybdate complex is reduced in the presence of phenolic compounds. A 100 *μ*L aliquot of each cork and corkback ethanol-water extract was added to 4 mL of Folin–Ciocalteu reagent and vortexed to homogenize the mixture. After 8 min at room temperature, 4 mL of 7.5% Na_2_CO_3_ solution was added and properly mixed. The mixture was incubated in a thermostatic bath at 45ºC for 15 min. The absorbance of the resulting blue colored mixtures was recorded in a spectrophotometer (UV-160A Recording Spectrophotometer, Shimadzu) at 765 nm against a blank containing only water. A gallic acid (GA) standard calibration curve (solutions within the range of concentrations 0–0.6 g/L) was used as a reference to measure the TPC. The experiment was conducted in triplicate, and the TPC was expressed as mg gallic acid equivalent (GAE) per g of extract (mean ± SD).

The total flavonoid content (TFC) was determined using a modified methodology described by Singleton et al. [28]. Aliquots of 1 mL of cork and corkback ethanol-water extracts were diluted in 4 mL of distilled water and 0.3 mL of 5% (m/v) NaNO_2_ was added. After 5 min in the dark, 0.3 mL of 10% (m/v) AlCl_3_ was added and, 6 min later, 2 mL of 4% (m/v) NaOH and 2.4 mL of distilled water was added sequentially and properly mixed. Absorbance of the mixture was measured at 506 nm after 30 min of incubation against water (UV-160A Recording Spectrophotometer, Shimadzu). A catechin (CE) standard curve (solutions within the range of concentrations 0.10–1.0 mg/mL) was used as reference to measure the TFC. The experiment was conducted in triplicate, and the TFC was expressed as mg catechin equivalent (CE) per g of extract (mean ± SD).

### 2.5. Antioxidant Activity

Two methods were used for the determination of the antioxidant activity of the ethanol-water extracts from the cork and corkback samples: 2,2-diphenyl-1-picryhydrazyl (DPPH) assay, which measures the free radical scavenging capacity, and ferric reducing antioxidant power (FRAP). It is important to carry out more than one type of antioxidant capacity measurement to cover the various mechanisms of antioxidant action of a plant extract [29].

#### 2.5.1. DPPH Assay

The DPPH assay was performed according to the method described by Abdulwahab et al. with some modifications [30]. It uses 2,2-diphenyl-1-picrylhydrazyl hydrate (DPPH), a nitrogen centered free radical having an odd electron which gives a strong absorption at 517 nm, and its color changes from purple to yellow when the odd electron is paired off in the presence of a radical scavenger to form the reduced DPPH-H [31]. Fresh DPPH solution was prepared by diluting 10 mL of stock solution (1 M) in 90 mL methanol. The DPPH results are expressed either as IC50 value or as Trolox equivalents on a dry extract base. Stock solutions of the cork and corkback ethanol-water extracts were prepared in the following concentrations: 0.27 mg/ml and 0.05 mg/ml for cork extracts from 1-2 mm and 0.25–0.45 mm fractions and 0.03 mg/ml and 0.07 mg/ml for corkback extracts from 1-2 mm and 0.25–0.45 mm fractions, respectively. Briefly, 1 mL of standard solutions and extracts was placed in test tubes and mixed with 3.9 mL of a DPPH methanol solution. The blank sample consisted of 1 mL of methanol added to 3.9 mL of DPPH solution. After 30 min incubation at room temperature in the dark, the absorbance was measured at 517 nm. Trolox is a water-soluble antioxidant, which was synthesized as a vitamin E derivative in 1974 [32]. Trolox has been used as a standard antioxidant for antioxidant capacity assays. The radical scavenging activity of each sample was expressed as IC50 (concentration of an inhibitor that results in a half-maximal inhibition of a response, i.e., the concentration that reduces a response to 50% of its maximum). The % inhibition of both standards and samples was calculated by the DPPH inhibition percentage as follows: I % = [(Abs_0_ − Abs_1_)/Abs_0_] × 100, where Abs_0_ was the absorbance of the blank and Abs_1_ was the absorbance of the sample at different concentrations and graphs were plotted (% inhibitions versus concentration). The scavenging effect of each extract on the DPPH radical was also expressed as the Trolox equivalent antioxidant capacity (TEAC) determined from the calibration curve with Trolox solution of different concentrations and the percentage of scavenging effect on the DPPH radical. Each experiment was carried out in triplicate.

#### 2.5.2. Ferric Reducing Antioxidant Power Assay

The ferric reducing antioxidant power (FRAP) assay depends on the reduction of ferric ion into ferrous ion [33]. A fresh working solution of FRAP reagent was obtained by mixing 25 mL 300 mM sodium acetate buffer (pH 3.6), 2.5 mL 10 mM TPTZ solution, and 2.5 mL 20 mM FeCl_3_.6H_2_O solution and warming at 37 ºC before use. An aliquot (100 *μ*L) of the cork and corkback ethanol-water extracts or of the standard was then added to 2.7 mL of FRAP reagent and 270 *μ*L of distilled water and the reaction mixture was incubated at 37°C for 30 min in the dark. The absorbance of the colored ferrous tripyridyltriazine complex was measured at 593 nm in comparison with a blank. Trolox was used for positive control and results are expressed in *μ*g Trolox equivalent.

### 2.6. Acetylcholinesterase Activity

The acetylcholinesterase (AChE) activity was assayed following an adaptation of the spectrophotometric method reported by Ellman et al. [34]. The cuvette used as a blank to control for the nonenzymatic hydrolysis of acetylcholine contained a mixture of 500 *μ*L of 3 mM DTNB solution (in 0.1 M potassium phosphate pH 8), 100 *μ*L of 15 mM AChI (in water), 275 *μ*L of 0.1 M potassium phosphate pH 8, and 100 *μ*L of each cork and corkback ethanol-water extract solutions at the different concentrations evaluated (0.1 mg/mL, 0.5 mg/mL, 1.0 mg/mL, and 2.0 mg/mL). In the reaction cuvette, 25 *μ*L of buffer was replaced by AChE solution 0.16 U/mL. The resulting solutions were placed in a spectrophotometer. The thiocholine formed during the hydrolysis of acetylcholine reacts rapidly with DTNB and a yellow compound is formed. The reaction was monitored for 5 min at 405 nm and the absorbance registered every minute. Velocities of reaction were calculated. Enzyme activity was calculated as a percentage of the velocities compared to that of the assay using buffer solution instead of inhibitor (cork or corkback ethanol-water extracts). The assays were performed in triplicate.

## 3. Results and Discussion

### 3.1. Total Phenolic and Total Flavonoid Contents

Table 1 presents the extraction yields, TPC, and TFC of the ethanol-water extracts of the cork and corkback samples with granulometry of 2-1 mm and 0.45–0.25 mm.

The yields of the lipophilic extracts of cork were similar for both granulometric fractions (on average 6.5%), in line with the 5-8% values reported by Pereira [23] for *Q. suber* cork, and averaged 2.7% for corkback, which is lower than the 4.4% reported by Pereira and Baptista [35].

The ethanol-water extraction yields of cork (1.8% and 4.0%) were below the range reported in the literature in ethanol-water extraction (varying between 7.1% and 8.3% according to Aroso et al. [36]. In corkback samples, the coarse fraction showed a high extraction yield (around 8%), while the fine fractions only yielded 1.5%. The differences in granulometric fraction yields are expected since, upon grinding, these fractions show specific structural features [37, 38]. In the case of cork, materials other than the cork cells may be present, e.g., lignified sclereids or lenticular filling material that will preferentially be present in the finer fractions thereby leading to chemical differences [37]. The influence of time and solvent's volume on the extraction yield was not evaluated in this study.

The total phenolic contents in the ethanol-water extracts of corkback and cork samples are in the range of 387.4–435.1 mg GAE/g extract and 189.9–202.9 mg GAE/g extract, correspondingly. When expressed in g GAE/kg of tissue, the total phenolic content ranged from 3.6 to 7.6 g GAE/kg of dry cork which is in line with those obtained in several other plant materials, but it was significantly higher for the corkback tissue (5.8–36.1 g GAE/kg of dry corkback) [25, 39, 40]. The TPC did not differ significantly between granulometric fractions, although it is considerably higher in corkback than in cork tissues.

TFC are also remarkably higher in corkback extracts (127.4–241.4 mg CE/g extract) in relation to the cork extracts. These differences were to be expected given the different anatomical origin and chemical composition of both materials. The corkback layer is of phloemic nature, constituted by a layer of nonconducting phloemic tissue that was isolated by the formation of an underlying periderm [26, 41].

### 3.2. Antioxidant Activity

The antioxidant activity of the ethanol-water extracts from cork and corkback was evaluated by DPPH and FRAP tests. Table 1 shows the antioxidant activity of the studied extracts, expressed in terms of the amount of extract required to reduce into 50% the DPPH concentration (IC50), as well as in terms of the Trolox equivalent antioxidant activity (TEAC) on an extract cork/corkback basis (mg TEAC/g extract). For FRAP assay, results are expressed in mM TEAC/g extract and mg Fe^2+^/g extract. The IC50 value for Trolox was also obtained and reported for comparative purposes (IC50 Trolox in ethanol 3.2 *μ*g Trolox/mL).

Plant polyphenols can act as free radical scavengers, especially flavonoids that may be potent antioxidants and applied against several diseases. Their specific chemical structure determines the ability of capturing the harmful free radicals [42]. To our knowledge, little is known about the antioxidant activity of corkback extracts. Our first observations give us the idea that particle size has no clear influence on the antiradical or reducing ability properties of extracts. Overall, both cork and corkback extracts showed good antioxidant properties and no evident differences were found between the two granulometric fractions. These ethanol-water extracts have revealed an antioxidant activity quite similar to the Trolox standard in terms of IC50; in fact, cork extracts have an IC50 similar to Trolox (3.2 *μ*g extract/mL) and a little higher for corkback extracts (4.0–5.3 *μ*g extract/mL). These values are in line with the results reported by Santos et al. [25] for *Q. suber* cork extracts of different ethanol-water polarity (2.79 in water extract, 3.58 in methanol, and 5.84 in methanol-water 50 : 50 extract).

These results are promising since cork and corkback polar (ethanol-water) extracts can be used as a source of potential natural antioxidants in nutraceutical applications or material surfacing protection.

### 3.3. Acetylcholinesterase Activity

**Figure 2:**
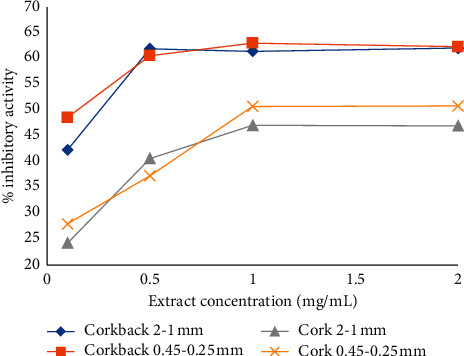
Influence of the cork and corkback ethanol-water extracts concentration on acetylcholinesterase inhibition.

In this study, the AChE inhibitory activity of the extracts expressed as IC_50_ values, calculated from the regression equations obtained from the activity of samples at different concentrations, was found to increase dose-dependently (Figure 2).

Overall, the corkback ethanol-water extracts showed higher inhibitory activity at all assayed concentrations (0.1–2.0 mg/mL) when compared to cork extracts, being superior in the ones with lower particle sizes. The same tendency was observed for the cork extracts.

At the highest assayed dose (2.0 mg/mL), the percent inhibitory effect was on average 62.2% in both granulometric fractions of corkback extracts, showing no significant differences between them. In fact, for concentrations equal to or higher than 0.5 mg/mL, the acetylcholinesterase inhibitory activity was always higher than 60% for corkback extracts, with no considerable increase above 1.0 mg/mL. However, at the lower assayed dose (0.1 mg/mL) the inhibitory effect was considerably lower, ranging from 42.4% to 48.6%.

The ethanol-water cork extracts showed an overall lower inhibitory effect, being around 48.9% at the higher assayed dose (2.0 mg/mL) and 26.2% at the lower assayed dose (0.1 mg/mL). The ethanol-water cork extracts displayed a nearly constant acetylcholinesterase inhibitory activity of 47.0% and 50.8% with extract concentrations equal to or greater than 1.0 mg/mL for cork particles of 2-1 mm and 0.45–0.25 mm, respectively (Figure 2).

Our results are within the range of the values found for the inhibition of this enzyme by plant extracts [43]. In fact, the results obtained for corkback extracts were better than the ones found for the standard galantamine (48.8% at 1.0 mg/mL) [44]. Galantamine is usually extracted from various species of Amaryllidaceae family as snowdrop, daffodil, and snowflake, which are scarce and threatened botanical sources supplies [45]. Extraction procedures, isolation, and purification steps are rather time consuming and highly costly, and therefore several synthetic protocols have been developed over the years, resulting in methodologies that can prepare galantamine in a few steps, in racemic or in enantiomerically pure form. However, these methodologies involve the use of toxic solvents and reagents and the yields of the resulting product range from low to moderate [45]. The major advantage on using cork and corkback extracts as AChE inhibitors relies on the fact that these raw materials are readily available residues of cork industry, contributing to a circular bioeconomy in which waste is converted to added-value products, supporting some of the 2030 agenda goals for sustainable development which supports rural activities' development, economic growth, social inclusion, and environmental protection. [46].

**Figure 3:**
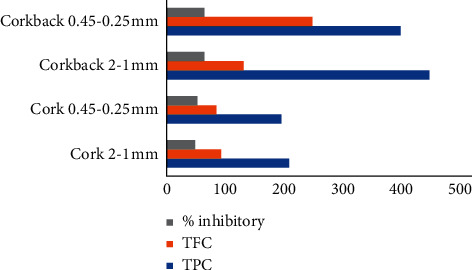
Correlation between the TPC and TFC with the inhibitory activity against AChE.

When comparing the acetylcholinesterase inhibitory activity with the overall phenolic and flavonoid contents of the extracts, we can see a positive direct correlation between them (Figure 3), as usually expected for plant extracts since these compounds have been previously reported to be potent agents for treating and combatting Alzheimer's disease [47, 48]. However, concerning the differences between granulometric fractions, TPC and TFC are higher in coarser fractions, whereas acetylcholinesterase inhibitory activity is smaller.

The results obtained are promising for both cork and corkback ethanol-water extracts as inhibitors of the acetylcholinesterase enzyme.

## 4. Conclusions

To our knowledge, this is the first report characterizing acetylcholinesterase inhibition together with antioxidant activities and phenolic contents of *Q. suber* cork and corkback ethanol-water extracts. These extracts show significant antioxidant activity against DPPH and FRAP, with a positive correlation between the phenolic and flavonoid contents. Corkback ethanol-water extracts, and cork extracts to a smaller extent, also showed an interesting inhibitory activity against acetylcholinesterase enzyme, resulting in their possible valorization as an accessible source of natural antioxidants and antiacetylcholinesterase agents in pharmaceutical or nutraceutical formulations with health benefits.

## Figures and Tables

**Table 1:** Lipophilic and polar extracts yields, composition in terms of total phenolic and total flavonoid contents, and antioxidant activity of ethanol-water extracts of cork and corkback samples from *Quercus suber* with particle sizes of 2-1 mm and 0.45–0.25 mm.

	Corkback		Cork	
	2-1 mm	0.45–0.25 mm	2-1 mm	0.45–0.25 mm
Extraction yield of CH_2_Cl_2_ extract (%)	1.8 ± 0.1	3.6 ± 0.2	6.3 ± 1.0	6.6 ± 0.4
Extraction yield of EtOH-water extract (%)	8.3 ± 0.2	1.5 ± 0.1	1.8 ± 0.4	4.0 ± 0.1
Total phenolic (mg GAE/g extract)	435.1 ± 8.1	387.4 ± 11.1	202.9 ± 10.2	189.9 ± 19.6
Total flavonoid (mg CE/g extract)	127.4 ± 77.5	241.4 ± 84.3	90.0 ± 30.4	82.4 ± 8.7
Antioxidant capacity (mg TEAC/g extract)	57.4 ± 1.30	66.3 ± 0.1	19.6 ± 1.6	21.0 ± 0.2
IC50 values (*μ*g extract/mL)^*∗*^	4.0 ± 0.1	5.2 ± 0.0	3.2 ± 0.1	3.2 ± 0.4
FRAP (mM TEAC/g extract)	9.2 ± 0.1	8.2 ± 0.2	3.1 ± 0.1	3.0 ± 0.1
FRAP (mg Fe^2+^/g extract)	9222.4 ± 36.3	8120.5 ± 27.2	2995.3 ± 19.6	2991.9 ± 41.6

GAE: gallic acid equivalent; CE: catechin equivalent; TEAC: Trolox equivalent antioxidant capacity; FRAP: ferric ion reducing antioxidant potential.

## Data Availability

All experimental data used to support the findings of this study are completely available from the corresponding author upon request.

## Abbreviations

AChE: Acetylcholinesterase
ACh: Acetylcholine
AD: Alzheimer's disease
C: Catechin
CE: Catechin equivalents
DPPH: 2,2-Diphenyl-1-picrylhydrazyl radical
DTNB: Ellman's reagent (5,5′-dithio-bis-[2-nitrobenzoic acid])
FRAP: Ferric ion reducing antioxidant potential
GA: Gallic acid
GAE: Gallic acid equivalents
GC-MS: Gas chromatography coupled with mass spectrometry
IC50: Half-maximal inhibitory concentration
SD: Standard deviation
TEAC: Trolox equivalent antioxidant capacity
TFC: Total flavonoid content
TPC: Total phenolic content
Trolox: 6-Hydroxy-2,5,7,8-tetramethylchroman-2-carboxylic acid
TPTZ: 2,4,6-Tri(2-pyridyl)-s-triazine.
